# Towards Simultaneous Actuator and Sensor Faults Estimation for a Class of Takagi-Sugeno Fuzzy Systems: A Twin-Rotor System Application

**DOI:** 10.3390/s20123486

**Published:** 2020-06-19

**Authors:** Marcin Pazera, Marcin Witczak, Norbert Kukurowski, Mariusz Buciakowski

**Affiliations:** Institute of Control and Computation Engineering, University of Zielona Góra, ul. Szafrana 2, 65-516 Zielona Góra, Poland; m.pazera@issi.uz.zgora.pl (M.P.); m.witczak@issi.uz.zgora.pl (M.W.); n.kukurowski@issi.uz.zgora.pl (N.K.)

**Keywords:** fault detection and diagnosis, faults estimation, actuator and sensor fault, observer design, Takagi-Sugeno fuzzy systems

## Abstract

The paper is devoted to the problem of estimating simultaneously states, as well as actuator and sensor faults for Takagi–Sugeno systems. The proposed scheme is intended to cope with multiple sensor and actuator faults. To achieve such a goal, the original Takagi–Sugeno system is transformed into a descriptor one containing all state and fault variables within an extended state vector. Moreover, to facilitate the overall design procedure an auxiliary fault vector is introduced. In comparison to the approaches proposed in the literature, a usual restrictive assumption concerning fixed fault rate of change is removed. Finally, the robust convergence of the whole observer is guaranteed by the so-called quadratic boundedness approach which assumes that process and measurement uncertainties are unknown but bounded within an ellipsoid. The last part of the paper portrays an exemplary application concerning a nonlinear twin-rotor system.

## 1. Introduction

Nowadays, owing to the interest in highly efficient systems, industrial companies permanently extend the number of sensors and actuators being used. Indeed, with the advent of IoT the number of components is systematically proliferating. This is mainly due to the fact of their relative low cost and wide accessibility. Irrespective of such an appealing effect, such a growth can increase the chance that actuator and sensor faults appear simultaneously. Moreover, the probability of multiple actuator and sensor faults is also increased. Thus, fault estimation is an important actor in modern Fault Diagnosis (FD) [1,2,3,4]. Indeed, it may give a knowledge about the presence, location and size of the fault. Such information is also a necessary ingredient of the active Fault-Tolerant Control (FTC) [5,6,7,8], which can accommodate the fault using appropriate control-oriented recovery actions.

There is no doubt that the problem of fault estimation was approached from many different angles. However, most of them focus on estimating either actuator or sensor faults. For a good representative example of recent developments in the area of actuator fault estimation the reader is referred to [9,10]. The case of sensor fault estimation can be realized similarly (cf. Pazera et al. [6] and the references therein).

The number of observers for simultaneous actuator and sensor faults is proliferating as well. However, most of them suffer due to the assumption of a limited fault rate, i.e., the fault derivative (or difference in consecutive discrete samples) is assumed to be close to zero.

Among the existing fault estimation strategies, the crucial attention is focused on the ones based on the sliding-mode observers [11,12], as well as the Kalman filter [13,14] and the related applications.

Taking into account the above discussion, it is also natural that the problem of simultaneous actuator and sensor fault estimation receives growing research attention [8,15,16,17,18]. However, the researches are developed for linear systems while the number of strategies capable of handling some classes of nonlinear systems is rather limited. Indeed, a recent state-of-the-art overview clearly indicates the trends for handling multiple simultaneous actuator and sensor fault estimation for nonlinear systems. Indeed, Gu et al. [19] converted an original Lipschitz system into a Linear Parameter Varying (LPV) one by a suitable reformulation of the Lipschitz property. The resulting design approach provides optimal fault estimates according to $H_{\infty }$ criterion within the frequency domain. Another appealing strategy for Lipschitz class of systems was introduced in Abdollahi [20]. It transforms the system into two subsystems while each of them is affected by either a sensor or an actuator fault, respectively. Finally, two separate Sliding Mode Observers (SMOs) are designed. This scheme suffers from the fact that an asymptotically convergence is guaranteed without considering any robustness to modelling uncertainities and/or external disturbances. A similar SMO was also developed in Hmidi et al. [21] and the authors remove the above drawback by ensuring robustness to bounded disturbances.

An important group of strategies convert the original nonlinear system into an equivalent Takagi-Sugeno one. A representative example of such strategies is presented in Bounemeur et al. [22]. As a result, an adaptive fuzzy estimator is developed capable of handling numerous fault scenarios. Unfortunately, it is devoted to deterministic systems neglecting uncertainty factors. Another strategy is developed in Fu et al. [23] and tackles the systems with switching non-linearities. Unfortunately, it also does not provide appropriate means for settling the robustness issue. Finally, in Shaker [24] a novel multiple integral unknown input observer is developed that is capable of decoupling disturbances. The final group of approaches are the ones for polynomial systems [25,26]. In this case, the design methodology reduces to converting the system by augmenting the state vector with fault variables. This strategy can also cope with process disturbances.

The unappealing feature of the existing approaches to simultaneous actuator and sensor fault estimation is that they do not provide sufficient information about the estimation quality. To handle this issue, two kind of approaches can be utilized: the first one uses post-fault measurements [27] and the second predicts the fault based on its historical values and past measurements [28]. It should be noted that the latter is the only scheme which can be applied in the active FTC. Unfortunately, such schemes struggle with the problem of minimising the effect of fault prediction [9,28].

The scheme proposed in this paper can handle the above mentioned issues, which constitutes the novelty of the proposed approach:

- the problem of one-step fault prediction and the related fault rate of change is removed by transforming the system into the descriptor Takagi-Sugeno one whose state vector contains both original states and the faults.
- the original system with the actuator fault with a time-varying distribution matrix is transformed into an equivalent one with a constant distribution matrix and the so-called auxiliary fault vector. This strategy naturally reduces the design conservatives.
- the effect of external disturbances is tackled with the so-called quadratic boundedness. As a result, the estimation quality can be assessed by the so-called uncertainty intervals of both states and faults. Thus, they can be perceived as possible worst cases of the unknown faults and states.

The paper is organised as follows. In Section 2, a simultaneous estimation of actuator and sensor faults problem is described along with suitable feasibility assumptions. Furthermore, a suitable convergence analysis is performed. Section 3 exhibits an illustrative example pertaining the twin-rotor system. Finally, Section 4 concludes the paper.

## 2. Observer Design

Let us start from the formulation of a Takagi-Sugeno (T-S) fuzzy system:

(1) $$\begin{array}{c} \begin{array}{cc} x_{k+1} & =A\left(v_{k}\right)x_{k}+B\left(v_{k}\right)u_{k}+B_{f}\left(v_{k}\right){\bar{f}}_{a,k}+W_{1}w_{1,k}\\ \; & =\sum_{i=1}^{M}h_{i}\left(v_{k}\right)\left[A^{i}x_{k}+B^{i}u_{k}+B_{f}^{i}{\bar{f}}_{a,k}\right]+W_{1}w_{1,k}, \end{array} \end{array}$$

(2) $$\begin{array}{cc} y_{k} & =Cx_{k}+C_{f}f_{s,k}+W_{2}w_{2,k}, \end{array}$$

with

(3) $$h_{i}\left(v_{k}\right)\geq 0,\;\forall i=1,\ldots ,M_{m},\;\;\sum_{i=1}^{M_{m}}h_{i}\left(v_{k}\right)=1,$$

where *k* stands for the discrete time and $x_{k}\in X\subset R^{n}$, $u_{k}\in R^{r}$, $y_{k}\in R^{m}$ are state, input and output vectors, respectively. Furthermore, ${\bar{f}}_{a,k}\in F_{a}\subset R^{n_{a}}$ and $f_{s,k}\in F_{s}\subset R^{n_{s}}$ signify the actuator and sensor fault vectors, respectively, where $n_{a}$ and $n_{s}$ stand for the number of actuator and sensor faults, respectively. Matrices A, B and C are the known state, input and output matrices, respectively, while $M_{m}$ stands for the number of submodels. Thus, $C_{f}$ denotes the sensor fault distribution matrix, where $\mathrm{rank}\left(C_{f}\right)=n_{s}$ and $\mathrm{rank}\left(B_{f}\left(v_{k}\right)\right)=n_{a}$ satisfy the inequality $n_{a}+n_{s}\leq m$. This means that it is impossible to estimate more faults than there are measured outputs. Finally, $W_{1}$ and $W_{2}$ are the distribution matrices of $w_{1,k}$ and $w_{2,k}$, which are exogenous disturbance vectors for the process and measurement uncertainties, respectively. The activation functions $h_{i}\left(\cdot \right)$ depend on the vector of premise variables $v_{k}={\left[v_{k}^{1},\;v_{k}^{2},\;\ldots ,\;v_{k}^{p}\right]}^{T}$, which is assumed to depend on measurable variables, e.g., system outputs and known inputs [29]. However, an extension towards unmeasurable premise variables is possible via direct applications of the solution proposed, e.g., in Ichalal et al. [30].

Finally, in the remaining part of the paper, the following notation is used $X\left(v_{k}\right)=\sum_{i=1}^{M}h_{i}\left(v_{k}\right)X^{i}$. First, let us start with transforming the state Equation (1) into an equivalent form

(4) $$\begin{array}{c} x_{k+1}=\sum_{i=1}^{M}h_{i}\left(v_{k}\right)\left[A^{i}x_{k}+B^{i}u_{k}\right]+B_{f}f_{a,k}+W_{1}w_{1,k}, \end{array}$$

with an auxiliary matrix $B_{f}$ satisfying $\mathrm{rank}\left(B_{f}\right)=n_{a}$ and an auxiliary actuator fault vector $f_{a,k}$. Comparing Equation (1) and Equation (4), it can be observed that

(5) $$\begin{array}{c} B_{f}\left(v_{k}\right){\bar{f}}_{a,k}=B_{f}f_{a,k}. \end{array}$$

Thus, having $f_{k}$, the original fault vector can be determined with

(6) $$\begin{array}{c} {\bar{f}}_{a,k}={\left(B_{f}\left(v_{k}\right)\right)}^{†}B_{f}f_{a,k}. \end{array}$$

where ${}^{†}$ stands for the pseudo inverse operator. Thus, the objective of further deliberations is to propose a novel observer capable of providing $x_{k}$, $f_{a,k}$ and $f_{s,k}$ estimates simultaneously. It should be also noted that the selection of $B_{f}$ is not critical, i.e., it can be set as $B_{f}=\frac{1}{M}\sum_{i=1}^{M}B_{f}^{i}$, which clearly preserves $\mathrm{rank}\left(B_{f}\right)=n_{a}$.

The proposed strategy starts with transforming Equation (4) and Equation (2) into an equivalent descriptor form with the following state variable

(7) $$\begin{array}{c} {\bar{x}}_{k}={\left[\begin{array}{ccc} x_{k}^{T} & f_{a,k-1}^{T} & f_{s,k}^{T} \end{array}\right]}^{T}. \end{array}$$

Using the above state vector Equation (7), the system Equation (4) and Equation (2) can be rewritten as:

(8) $$\begin{array}{cc} E{\bar{x}}_{k+1} & =\bar{A}\left(v_{k}\right){\bar{x}}_{k}+\bar{B}\left(v_{k}\right)u_{k}+{\bar{W}}_{1}{\bar{w}}_{k}, \end{array}$$

(9) $$\begin{array}{cc} y_{k} & =\bar{C}x_{k}+{\bar{W}}_{2}{\bar{w}}_{k}, \end{array}$$

with:

$$\begin{array}{cc} E & =\left[\begin{array}{ccc} I_{n} & -B_{f} & 0\\ 0 & 0 & 0\\ 0 & 0 & 0 \end{array}\right],\;\bar{A}\left(v_{k}\right)=\left[\begin{array}{ccc} A\left(v_{k}\right) & 0 & 0\\ 0 & 0 & 0\\ 0 & 0 & 0 \end{array}\right],\;\bar{B}\left(v_{k}\right)=\left[\begin{array}{c} B\left(v_{k}\right)\\ 0\\ 0 \end{array}\right],\\ {\bar{W}}_{1} & =\left[\begin{array}{cc} W_{1} & 0\\ 0 & 0\\ 0 & 0 \end{array}\right],\;{\bar{W}}_{2}=\left[\begin{array}{cc} 0 & W_{2} \end{array}\right],\;{\bar{w}}_{k}={\left[\begin{array}{cc} w_{1,k}^{T} & w_{2,k}^{T} \end{array}\right]}^{T},\;\bar{C}=\left[\begin{array}{ccc} C & 0 & C_{f} \end{array}\right]. \end{array}$$

This means that both faults as well as the state of the system are incorporated into a super state vector ${\bar{x}}_{k}$. Thus, for the purpose of the state estimation obeying Equation (8) and Equation (9), the following observer is proposed:

(10) $$\begin{array}{cc} z_{k+1} & =N\left(v_{k}\right)z_{k}+M\left(v_{k}\right)u_{k}+L\left(v_{k}\right)y_{k}, \end{array}$$

(11) $$\begin{array}{cc} {\hat{\bar{x}}}_{k} & =z_{k}+T_{2}y_{k}, \end{array}$$

where $z_{k}\in R^{n+na+ns}$ is the internal state of the estimator while ${\hat{\bar{x}}}_{k}\in R^{n+na+ns}$ stands for the estimate of Equation (7).

It should be noted that the proposed approach eliminates the usual assumption concerning a bounded rate of change of actuator and sensor fault, which increase the conservatives level of the approaches proposed in the literature (see, e.g., Pazera et al. [28] and the references therein.)

Let us start with assuming that there exist matrices $T_{1}$ and $T_{2}$ such that

(12) $$T_{1}E+T_{2}\bar{C}=I,$$

or

(13) $$\begin{array}{c} \left[\begin{array}{cc} T_{1} & T_{2} \end{array}\right]\left[\begin{array}{c} E\\ \bar{C} \end{array}\right]=I, \end{array}$$

which yields Equation (10) and Equation (11) design condition

(14) $$\mathrm{rank}\left(\left[\begin{array}{c} E\\ \bar{C} \end{array}\right]\right)=n+n_{a}+ns.$$

Under the above assumption, it is possible to derive the error of state estimation with Equation (11), i.e.,

(15) $$\begin{array}{c} \begin{array}{c} e_{k}={\bar{x}}_{k}-{\hat{\bar{x}}}_{k}={\bar{x}}_{k}-z_{k}-T_{2}y_{k}=\left(I-T_{2}\bar{C}\right){\bar{x}}_{k}-z_{k}-T_{2}{\bar{W}}_{2}{\bar{w}}_{k}, \end{array} \end{array}$$

which, by using Equation (12), boils down to

(16) $$\begin{array}{c} e_{k}=T_{1}E{\bar{x}}_{k}-z_{k}-T_{2}{\bar{W}}_{2}{\bar{w}}_{k}. \end{array}$$

Thus, by substituting Equations (8)–(10), the dynamics of state estimation error obeys

(17) $$\begin{array}{c} \begin{array}{cc} e_{k+1} & =T_{1}E{\bar{x}}_{k+1}-z_{k+1}-T_{2}{\bar{W}}_{2}{\bar{w}}_{k+1}=T_{1}\bar{A}\left(v_{k}\right){\bar{x}}_{k}+T_{1}\bar{B}\left(v_{k}\right)u_{k}\\ \; & +T_{1}{\bar{W}}_{1}{\bar{w}}_{k}-N\left(v_{k}\right)z_{k}-M\left(v_{k}\right)u_{k}-L\left(v_{k}\right)\bar{C}{\bar{x}}_{k}-L\left(v_{k}\right){\bar{W}}_{2}{\bar{w}}_{k}-T_{2}{\bar{W}}_{2}{\bar{w}}_{k+1}\\ \; & =\left(T_{1}\bar{A}\left(v_{k}\right)-L\left(v_{k}\right)\bar{C}-\left(v_{k}\right)T_{1}E\right){\bar{x}}_{k}+\left(T_{1}\bar{B}\left(v_{k}\right)-M\left(v_{k}\right)\right)u_{k}\\ \; & +N\left(v_{k}\right)e_{k}+\left(T_{1}{\bar{W}}_{1}-L\left(v_{k}\right){\bar{W}}_{2}+N\left(v_{k}\right)T_{2}{\bar{W}}_{2}\right){\bar{w}}_{k}-T_{2}{\bar{W}}_{2}{\bar{w}}_{k+1}. \end{array} \end{array}$$

From Equation (17), it is evident that the following relations should be satisfied:

(18) $$\begin{array}{cc} T_{1}\bar{A}\left(v_{k}\right)-N\left(v_{k}\right)T_{1}E-L\left(v_{k}\right)\bar{C} & =0, \end{array}$$

(19) $$\begin{array}{cc} T_{1}\bar{B}\left(v_{k}\right)-M\left(v_{k}\right) & =0. \end{array}$$

Indeed, by satisfying Equation (18), the term related to ${\bar{x}}_{k}$ vanished from Equation (17). Similarly, satisfying Equationv Equation (19) means that Equation (18) is no longer dependent on the system input $u_{k}$. Applying Equation (12) to Equation (18) gives

(20) $$T_{1}\bar{A}\left(v_{k}\right)-N\left(v_{k}\right)\left(I-T_{2}\bar{C}\right)-L\left(v_{k}\right)\bar{C}=0,$$

or

(21) $$N\left(v_{k}\right)=T_{1}\bar{A}\left(v_{k}\right)-\left(L\left(v_{k}\right)-N\left(v_{k}\right)T_{2}\right)\bar{C}.$$

Finally, by defining

(22) $$K\left(v_{k}\right)=L\left(v_{k}\right)-N\left(v_{k}\right)T_{2},$$

equality Equation (21) boils down to

(23) $$N\left(v_{k}\right)=T_{1}\bar{A}\left(v_{k}\right)-K\left(v_{k}\right)\bar{C},$$

which makes it possible to transform Equation (17) into

(24) $$\begin{array}{c} \begin{array}{cc} e_{k+1} & =N\left(v_{k}\right)e_{k}+\left(T_{1}{\bar{W}}_{1}-L\left(v_{k}\right){\bar{W}}_{2}+N\left(v_{k}\right)T_{2}{\bar{W}}_{2}\right){\bar{w}}_{k}-T_{2}{\bar{W}}_{2}{\bar{w}}_{k+1}\\ \; & =\left(T_{1}\bar{A}\left(v_{k}\right)-K\left(v_{k}\right)\bar{C}\right)e_{k}+T_{1}{\bar{W}}_{1}{\bar{w}}_{k}-K\left(v_{k}\right){\bar{W}}_{2}{\bar{w}}_{k}-N\left(v_{k}\right)T_{2}{\bar{W}}_{2}{\bar{w}}_{k}\\ \; & +N\left(v_{k}\right)T_{2}{\bar{W}}_{2}{\bar{w}}_{k}-T_{2}{\bar{W}}_{2}{\bar{w}}_{k+1}=\left(T_{1}\bar{A}\left(v_{k}\right)-K\left(v_{k}\right)\bar{C}\right)e_{k}\\ \; & +T_{1}{\bar{W}}_{1}{\bar{w}}_{k}-K\left(v_{k}\right){\bar{W}}_{2}{\bar{w}}_{k}-T_{2}{\bar{W}}_{2}{\bar{w}}_{k+1}. \end{array} \end{array}$$

Subsequently, by defining the following super-vector

(25) $$\begin{array}{c} {\tilde{w}}_{k}=\left[\begin{array}{c} {\bar{w}}_{k}\\ {\bar{w}}_{k+1} \end{array}\right], \end{array}$$

equality Equation (24) can be rewritten into a simpler form

(26) $$\begin{array}{c} e_{k+1}=X\left(v_{k}\right)e_{k}+Z\left(v_{k}\right){\tilde{w}}_{k}, \end{array}$$

with:

$$\begin{array}{c} X\left(v_{k}\right)=\tilde{A}\left(v_{k}\right)-K\left(v_{k}\right)\bar{C},\;Z\left(v_{k}\right)={\tilde{W}}_{1}-K\left(v_{k}\right){\tilde{W}}_{2}, \end{array}$$

where:

$$\begin{array}{c} {\tilde{W}}_{1}\left(v_{k}\right)=\left[\begin{array}{cc} T_{1}{\bar{W}}_{1} & -T_{2}{\bar{W}}_{2} \end{array}\right],\;{\tilde{W}}_{2}=\left[\begin{array}{cc} {\bar{W}}_{2} & 0 \end{array}\right],\;\tilde{A}\left(v_{k}\right)=T_{1}\bar{A}\left(v_{k}\right). \end{array}$$

For the purpose of further convergence analysis, let us start with reminding the Finsler’s Lemma [31]:

**Lemma** **1.** *The following expressions are equivalent:*

*1.*  ${\tilde{x}}_{k}^{T}Q{\tilde{x}}_{k}<0,\;\forall {\tilde{x}}_{}\in \left\{{\tilde{x}}_{}\in R^{n_{x}}|{\tilde{x}}_{}\neq 0,R{\tilde{x}}_{}=0\right\},$
*2.*  *$\exists \bar{M}\in R^{n+ns\times m}$ such that $Q+\bar{M}R+R^{T}{\bar{M}}^{T}<0$.*

Let us also define the Lyapunov function

(27) $$V_{k}=e_{k}^{T}Pe_{k},$$

with P≻0. Furthermore, the estimation error Equation (26) can be rewritten in an alternative form

(28) $$X\left(v_{k}\right)e_{k}+Z\left(v_{k}\right){\tilde{w}}_{k}-e_{k+1}=0,$$

which implies that the following extended-vector can be defined

(29) $${\tilde{x}}_{k}={\left[\begin{array}{ccc} e_{k}^{T} & {\tilde{w}}_{k}^{T} & e_{k+1}^{T} \end{array}\right]}^{T},$$

and as a consequence the following statement can be formulated

(30) $$R\left(v_{k}\right)\left[\begin{array}{c} e_{k}\\ {\tilde{w}}_{k}\\ e_{k+1} \end{array}\right]=0.$$

Thus, based on Equation (28), Equation (29) and Equation (30), it can be shown that

(31) $$R\left(v_{k}\right)=\left[X\left(v_{k}\right)\;\;Z\left(v_{k}\right)\;\;-I\right],$$

where

(32) $$R\left(v_{k}\right){\tilde{x}}_{k}=0,$$

in order to satisfy Equation (28). The convergence of the proposed observer is to be determined with the so-called Quadratic Boundedness (QB) approach [32]. This technique can be perceived as an extension of the usual Lyapunov approach towards the systems with external bounded disturbances. The usefulness of QB approach was proven in many papers while in Pazera et al. [28] it was proven that the standard $H_{\infty }$ framework can be perceived as a special case of QB. To use the QB approach, it is necessary to assume that ${\tilde{w}}_{k}$ is bounded by the following ellipsoid:

(33) $$\begin{array}{c} E_{w}=\left\{{\tilde{w}}_{k}:\;\;{\tilde{w}}_{k}^{T}Q_{w}{\tilde{w}}_{k}\leq 1\right\}, \end{array}$$

with $Q_{w}≻0$. Under such assumptions, the following definition can be recalled:

**Definition** **1.** The system Equation (26) is strictly quadratically bounded for all ${\tilde{w}}_{k}\in E_{w}$, k≥0, if $V_{k}>1\Rightarrow V_{k+1}-V_{k}<0$ for any ${\tilde{w}}_{k}\in E_{w}$.

As shown in Alessandri et al. [32] and Pazera et al. [28], the stability condition associated with

(34) $$\begin{array}{c} V_{k+1}-\left(1-\alpha \right)V_{k}-\alpha {\tilde{w}}_{k}^{T}Q_{w}{\tilde{w}}_{k}<0, \end{array}$$

with 0<α<1. Based on the above considerations, the following theorem is established:

**Theorem** **1.** *The observer-based system Equation (26) is strictly quadratically bounded for all ${\tilde{w}}_{k}\in E_{w}$ if there exist matrices P≻0, U, $\bar{N}$ as well as α∈(0,1) such that the following holds:*

(35) $$\left[\begin{array}{ccc} \alpha P-P & 0 & {\tilde{A}}^{T}\left(v_{k}\right)U^{T}-{\bar{C}}^{T}{\bar{N}}^{T}\left(v_{k}\right)\\ 0 & -\alpha Q_{w} & {\tilde{W}}_{1}^{T}U^{T}-{\tilde{W}}_{2}^{T}{\bar{N}}^{T}\left(v_{k}\right)\\ U\tilde{A}\left(v_{k}\right)-\bar{N}\left(v_{k}\right)\bar{C} & U{\tilde{W}}_{1}-\bar{N}\left(v_{k}\right){\tilde{W}}_{2} & P-U-U^{T} \end{array}\right]≺0,$$

**Proof.**  Using Equation (34) and setting

(36) $$\begin{array}{cc} Q & =\left[\begin{array}{ccc} \alpha P-P & 0 & 0\\ 0 & -\alpha Q_{w} & 0\\ 0 & 0 & P \end{array}\right], \end{array}$$

(37) $$\begin{array}{cc} \bar{M} & ={\left[\begin{array}{ccc} 0^{T} & 0^{T} & U^{T} \end{array}\right]}^{T}. \end{array}$$

along with Lemma 1 leads to

(38) $$\left[\begin{array}{ccc} \alpha P-P & 0 & X^{T}\left(v_{k}\right)U^{T}\\ 0 & -\alpha Q_{w} & Z^{T}\left(v_{k}\right)U^{T}\\ UX\left(v_{k}\right) & UZ\left(v_{k}\right) & P-U-U^{T} \end{array}\right]≺0.$$

Setting

(39) $$\begin{array}{cc} UX\left(v_{k}\right) & =U\left(\tilde{A}\left(v_{k}\right)-K\left(v_{k}\right)\bar{C}\right)=U\tilde{A}\left(v_{k}\right)-\bar{N}\left(v_{k}\right)\bar{C}, \end{array}$$

(40) $$\begin{array}{cc} UZ\left(v_{k}\right) & =U\left({\tilde{W}}_{1}-K\left(v_{k}\right){\tilde{W}}_{2}\right)=U{\tilde{W}}_{1}-\bar{N}\left(v_{k}\right){\tilde{W}}_{2}, \end{array}$$

into Equation (38) completes the proof. □

Irrespective of the incontestable appeal of the proposed solution Equation (35), its numerical tractability is feasible only if it is transformed to the set of linear matrix inequalities under fixed α:

(41) $$\left[\begin{array}{ccc} \alpha P-P & 0 & {{\tilde{A}}^{i}}^{T}U^{T}-{\bar{C}}^{T}{{\bar{N}}^{i}}^{T}\\ 0 & -\alpha Q_{w} & {\tilde{W}}_{1}^{T}U^{T}-{\tilde{W}}_{2}^{T}{{\bar{N}}^{i}}^{T}\\ U{\tilde{A}}^{i}-{\bar{N}}^{i}\bar{C} & U{\tilde{W}}_{1}-{\bar{N}}^{i}{\tilde{W}}_{2} & P-U-U^{T} \end{array}\right]≺0,\;i=1,\ldots ,M.$$

Finally, the design procedure boils down to:

**Step 0:**  Determine $T_{1}$ and $T_{2}$ by solving Equation (12).
**Step 1:**  Set α, 0<α<1 and determine ${\bar{N}}^{i}$ and U by solving Equation (41).
**Step 2:**  Calculate:
  (42) $$\begin{array}{cc} K^{i} & =U^{-1}{\bar{N}}^{i}, \end{array}$$
  (43) $$\begin{array}{cc} N^{i} & =T_{1}{\tilde{A}}^{i}-K^{i}\bar{C}, \end{array}$$
  (44) $$\begin{array}{cc} M^{i} & =T_{1}{\bar{B}}^{i}, \end{array}$$
  (45) $$\begin{array}{cc} L^{i} & =K^{i}+N^{i}T_{2}. \end{array}$$

While the application procedure leads to:

**Step 0:**  Substitute k=0 and set the initial conditions $z_{0}$.
**Step 1:**  Calculate $z_{k+1}$ and ${\hat{x}}_{k}$ with Equations (10) and (11).
**Step 2:**  Set k=k+1 and go to *Step 1*.

As demonstrated in Pazera et al. [28], the value of α has a direct influence of the convergence rate of the underlying observer. On the other hand, matrix P shapes the ellipsoidal bound of the estimation error:

(46) $$\begin{array}{c} e_{k}^{T}Pe_{k}\leq \zeta_{k},\;\zeta_{k}=1+{\left(1-\alpha \right)}^{k}\left(1-e_{0}^{T}Pe_{0}\right), \end{array}$$

which can be directly used to determine the uncertainty intervals of the state variables [28]:

(47) $$\begin{array}{c} {\hat{\bar{x}}}_{k,i}-\sqrt{\zeta_{k}c_{i}^{T}P^{-1}c_{i}}\leq {\bar{x}}_{k,i}\leq {\hat{\bar{x}}}_{k,i}+\sqrt{\zeta_{k}c_{i}^{T}P^{-1}c_{i}} \end{array}$$

where $c^{i}$ signifies *i*th column of an identity matrix of an appropriate dimension.

## 3. Case Study: Twin-Rotor System

**Figure 1:**
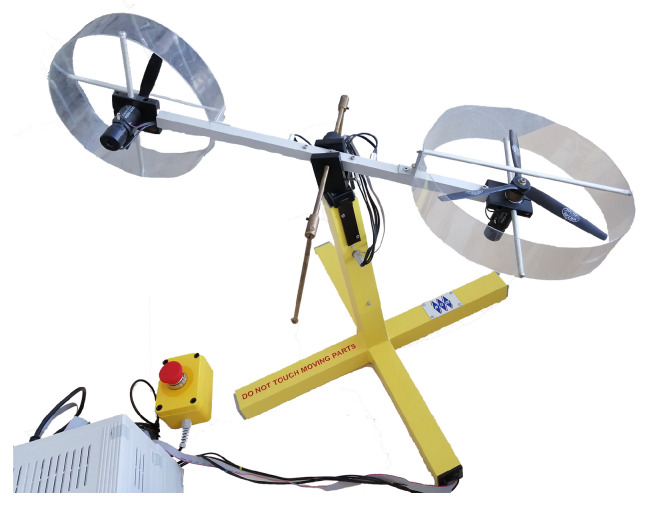
A nonlinear twin-rotor MIMO system.

The aim of this section is to validate the performance and correctness of the novel observer. Accordingly, the proposed actuator and sensor fault estimation method has been implemented to the Twin-Rotor System (TRS) [28], illustrated in Figure 1. Note that all equations and a detailed nonlinear model of the TRS can be found in [28]. This model was further transformed into the Takagi-Sugeno form using the dedicated methodology proposed in Rotondo et al. [33]. The matrices shaping the model Equation (1) and Equation (2) are presented in the Appendix A.

The state vector of the system is given by

(48) $$\begin{array}{c} x_{}={\left[\begin{array}{cccccc} \omega_{v} & \Omega_{v} & \theta_{v} & \omega_{h} & \Omega_{h} & \theta_{h} \end{array}\right]}^{T}, \end{array}$$

hence the input vector is described by

(49) $$\begin{array}{c} u_{}={\left[\begin{array}{cc} u_{v} & u_{h} \end{array}\right]}^{T}, \end{array}$$

where $\omega_{v}$ and $\Omega_{v}$ stand for the rotational and angular velocities of the main rotor while $\theta_{v}$ signifies the pitch angle of the beam. Furthermore, $\omega_{h}$ and $\Omega_{h}$ are the tail rotor rotational as well as angular velocities whilst $\theta_{h}$ is the yaw angle of the beam. Finally, $u_{v}$ and $u_{h}$ stand for actuation due to the main and tail DC motors, respectively. It should be noted that both inputs $u_{v}$ and $u_{h}$ operate in range (−1,1). Moreover, all details and parameters can be found in manufacturer’s user manual [34] and in Pazera et al. [28].

The following fault scenarios have been considered:

(50) $$\begin{array}{cc} f_{a,1,k} & =\left\{\begin{array}{cc} -0.1\cdot u_{1,k} & \;3000\leq k\leq 6500\\ 0 & \;\mathrm{otherwise}, \end{array}\right. \end{array}$$

(51) $$\begin{array}{cc} f_{a,2,k} & =\left\{\begin{array}{cc} 0.1\cdot u_{2,k} & \;4500\leq k\leq 8000\\ 0 & \;\mathrm{otherwise}, \end{array}\right. \end{array}$$

(52) $$\begin{array}{cc} f_{s,1,k} & =\left\{\begin{array}{cc} y_{k}-2.5 & \;5000\leq k\leq 7000\\ 0 & \;\mathrm{otherwise}, \end{array}\right. \end{array}$$

(53) $$\begin{array}{cc} f_{s,2,k} & =\left\{\begin{array}{cc} y_{k}+1.6 & \;6000\leq k\leq 9500\\ 0 & \;\mathrm{otherwise}. \end{array}\right. \end{array}$$

According to the approach proposed along with Equation (6):

(54) $$B_{f}=\frac{1}{M_{m}}\sum_{i=1}^{M_{m}}B_{f}^{i}=\left[\begin{array}{cc} 49.4084\cdot 10^{-6} & -66.2480\cdot 10^{-6}\\ 14.3375\cdot 10^{-3} & -13.2367\cdot 10^{-3}\\ 303.3781 & 0\\ 6.9349\cdot 10^{-6} & 3.6497\cdot 10^{-6}\\ 1.3860\cdot 10^{-3} & 1.0854\cdot 10^{-3}\\ 0 & 58.0016 \end{array}\right],$$

which means that all actuators are considered as possibly faulty and their faults have to be estimated. Whilst the sensor fault distribution matrix is obtained from matrix C (see Appendix A, Equation (A25)) by extracting the rows corresponding to the first and second sensor, respectively and it is given as follows

(55) $$C_{f}={\left[\begin{array}{ccccc} 0 & 0 & 1 & 0 & 0\\ 0 & 0 & 0 & 1 & 0 \end{array}\right]}^{T}.$$

**Figure 2:**
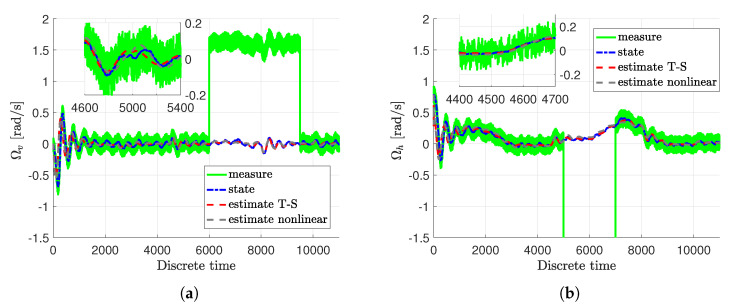
Comparison between nonlinear and Takagi-Sugeno response of the system: angular velocity of the main rotor $\Omega_{h}$ (**a**) and angular velocity of the tail rotor $\Omega_{v}$ (**b**).

It can be clearly viewed that within this scenario, both actuator and sensor faults are partially at the same time. Moreover, the considered faults are constant biases, which are either positive or negative. From Equations (50)–(53), it can be clearly viewed that actuator and sensor faults appear partially at the same time. The considered actuator fault Equation (50) pertains a 10% performance decrease of the first actuator, while Equation (51) concerns a 10% performance increase of the second actuator. Moreover, the considered sensor faults Equation (52) and Equation (53) are represented by either positive or negative constant biases equal to 1.6 or −2.5, respectively. Thus, they denote a significant sensor readings inaccuracies. However, the distribution matrix of sensor fault denotes that they influence the measurements of the angular velocity of the tail rotor $\Omega_{h}$ as well as the angular velocity of the main rotor $\Omega_{v}$. Firstly, let us present a comparison between nonlinear estimation approach described in Pazera et al. [28] and the proposed Takagi-Sugeno scheme, which is shown in Figure 2a,b. As it can be observed, the Takagi-Sugeno model-based approach follows the response of the original nonlinear model-based one, and hence, it does not impair the quality of state and fault estimates significantly.

**Figure 3:**
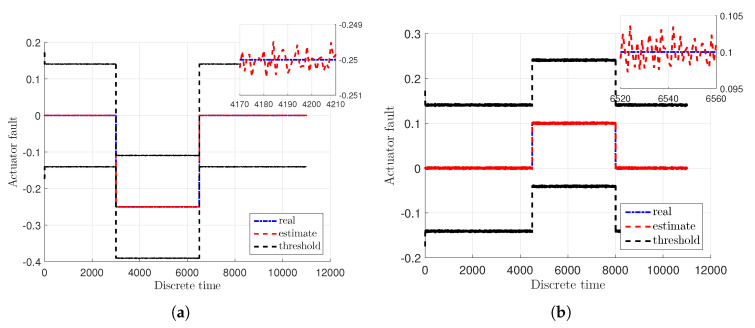
Actuator faults $f_{a,1}$ (**a**) and $f_{a,2}$ (**b**).

**Figure 4:**
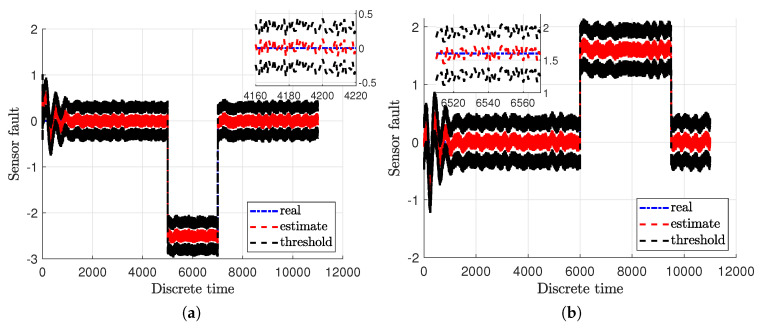
Sensor faults $f_{s,1}$ (**a**) and $f_{s,2}$ (**b**).

Figure 3a,b illustrate the real actuator faults of the main $f_{a,1}$ and tail $f_{a,2}$ rotor with blue dash-dotted lines along with their estimates given with red dashed lines. Additionally, the real and estimated values are overbounded by uncertainty intervals which are indicated with black dashed lines. Moreover, the real sensor faults $f_{s,1}$ and $f_{s,2}$ are presented in Figure 4a,b with blue dash-dotted lines as well as their estimates depicted by red dashed lines along with the uncertainty intervals indicated with black dashed lines. From these figures it is easily seen that the actuator and sensor faults were estimated with a very good accuracy under the measurement uncertainties. Consequently, the system states have been correctly reconstructed.

**Figure 5:**
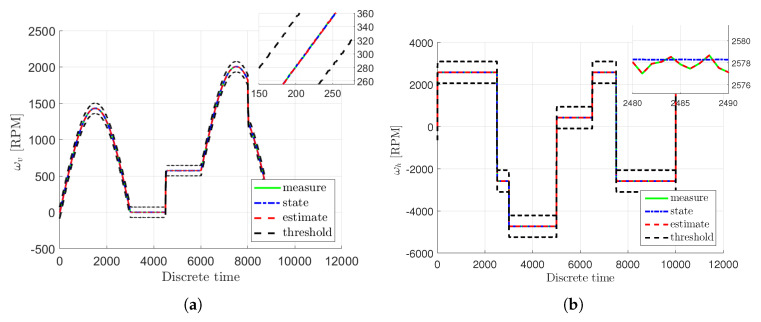
State variables $\omega_{v}$ (**a**) and $\omega_{h}$ (**b**).

**Figure 6:**
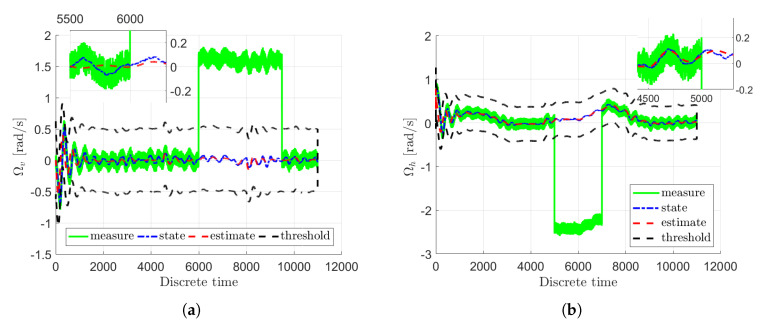
State variables $\Omega_{v}$ (**a**) and $\Omega_{h}$ (**b**).

**Figure 7:**
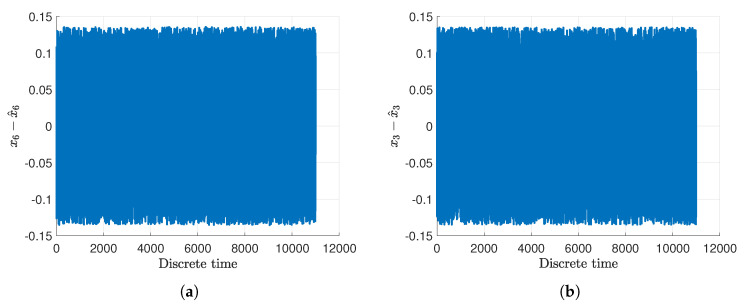
Evolution of the state estimation error for the rotational velocities of the main rotor (**a**) and of the tail rotor (**b**).

**Figure 8:**
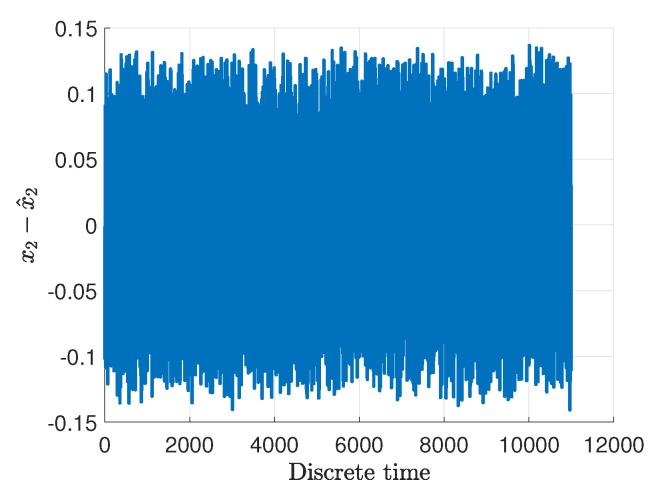
Evolution of the state estimation error for the pitch angle of the beam.

Figure 5a,b show the real rotational velocities of the main $\omega_{v}$ and tail $\omega_{h}$ rotor with blue dash-dotted lines. Their measured outputs are presented by light green solid lines along with their estimates given with red dashed lines as well as the uncertainty intervals indicated with black dashed lines. Moreover, Figure 6a,b present the angular velocities of the main $\Omega_{v}$ and of the tail $\Omega_{h}$ rotor with blue dash-dotted lines whilst their estimates are given with red dashed lines as well as the measured outputs depicted by light green solid lines. Additionally, the uncertainty intervals are indicated with black dashed lines. As can be observed in Figure 6a,b, irrespective of the intermittent fault (marked in light green) the state estimates are very close to the original states. The rotational velocities of the main and tail rotor and the pitch angle of the beam have been accurately estimated even if the actuator and sensor faults occurred simultaneously. This fact is illustrated in Figure 7a,b and Figure 8, which show the evolution of the state estimation error for the above variables. The figures clearly show that the states are properly reconstructed under the actuator glitch along with positive and negative incorrect readings of the sensor.

## 4. Concluding Remarks

An important aspect of the paper was to develop an observer for the Takagi-Sugeno systems which would be capable of estimating both state with both actuator and sensor. The first part of the proposed strategy was developed in such a way that no adaptive observer is required to obtain the sensor fault estimates. In other words, it is obtained directly by simply transforming an output equation. The second part of the observer concerns the adaptive actuator fault estimation and the state as well. In order to achieve robustness against process and measurement uncertainties and to ensure the stability performance a Quadratic Boundedness strategy is employed. For the purpose of obtaining the observer gain matrices, a set of LMIs is needed to be solved. The other part of the paper presents a performance validation. It has been achieved by implementing the approach to a nonlinear Twin-Rotor MIMO System. The obtained results plainly confirm that the desired properties of the observer have been accomplished. The state has been estimated properly even in the case of both the actuator and sensor fault case. While analysing an actuator fault, it can be concluded that its estimation error is at a very low level in both cases: fault and fault-free as well. Furthermore, the obtained sensor fault estimates quality strongly depends on the quality of the state estimation. The future work will be devoted to applying such an approach to a fault-tolerant control scheme for a T–S fuzzy systems. 

## Appendix A

(A1) $$\begin{array}{cc} A^{1} & =\left[\begin{array}{cccccc} 1 & 9.9709\cdot 10^{-3} & 4.5486\cdot 10^{-7} & -5.1930\cdot 10^{-7} & -1.7300\cdot 10^{-9} & -1.5629\cdot 10^{-14}\\ 0 & 9.9418\cdot 10^{-1} & 8.5060\cdot 10^{-5} & -1.0376\cdot 10^{-4} & -5.1856\cdot 10^{-7} & -6.2171\cdot 10^{-12}\\ 0 & 0 & 6.6291\cdot 10^{-1} & 0 & 0 & 0\\ 0 & 0 & 0 & 9.9988\cdot 10^{-1} & 9.9785\cdot 10^{-3} & 1.7793\cdot 10^{-7}\\ 0 & 0 & 0 & -2.4758\cdot 10^{-2} & 9.9566\cdot 10^{-1} & 3.4967\cdot 10^{-5}\\ 0 & 0 & 0 & 0 & 0 & 9.0333\cdot 10^{-1} \end{array}\right], \end{array}$$

(A2) $$\begin{array}{cc} A^{2} & =\left[\begin{array}{cccccc} 1 & 9.9709\cdot 10^{-3} & 4.5486\cdot 10^{-7} & -4.6737\cdot 10^{-7} & -1.5570\cdot 10^{-9} & -1.4066\cdot 10^{-14}\\ 0 & 9.9418\cdot 10^{-1} & 8.5060\cdot 10^{-5} & -9.3380\cdot 10^{-5} & -4.6671\cdot 10^{-7} & -5.5954\cdot 10^{-12}\\ 0 & 0 & 6.6291\cdot 10^{-1} & 0 & 0 & 0\\ 0 & 0 & 0 & 9.9988\cdot 10^{-1} & 9.9785\cdot 10^{-3} & 1.7793\cdot 10^{-7}\\ 0 & 0 & 0 & -2.4758\cdot 10^{-2} & 9.9566\cdot 10^{-1} & 3.4967\cdot 10^{-5}\\ 0 & 0 & 0 & 0 & 0 & 9.0333\cdot 10^{-1} \end{array}\right], \end{array}$$

(A3) $$\begin{array}{cc} A^{3} & =\left[\begin{array}{cccccc} 1 & 9.9707\cdot 10^{-3} & 4.5690\cdot 10^{-7} & -5.2162\cdot 10^{-7} & -1.7378\cdot 10^{-9} & -1.5699\cdot 10^{-14}\\ 0 & 9.9415\cdot 10^{-1} & 8.5440\cdot 10^{-5} & -1.0422\cdot 10^{-4} & -5.2088\cdot 10^{-7} & -6.2449\cdot 10^{-12}\\ 0 & 0 & 6.6291\cdot 10^{-1} & 0 & 0 & 0\\ 0 & 0 & 0 & 9.9988\cdot 10^{-1} & 9.9785\cdot 10^{-3} & 1.7793\cdot 10^{-7}\\ 0 & 0 & 0 & -2.4758\cdot 10^{-2} & 9.9566\cdot 10^{-1} & 3.4967\cdot 10^{-5}\\ 0 & 0 & 0 & 0 & 0 & 9.0333\cdot 10^{-1} \end{array}\right], \end{array}$$

(A4) $$\begin{array}{cc} A^{4} & =\left[\begin{array}{cccccc} 1 & 9.9707\cdot 10^{-3} & 4.5690\cdot 10^{-7} & -4.6946\cdot 10^{-7} & -1.5640\cdot 10^{-9} & -1.4129\cdot 10^{-14}\\ 0 & 9.9415\cdot 10^{-1} & 8.5440\cdot 10^{-5} & -9.3798\cdot 10^{-5} & -4.6879\cdot 10^{-7} & -5.6204\cdot 10^{-12}\\ 0 & 0 & 6.6291\cdot 10^{-1} & 0 & 0 & 0\\ 0 & 0 & 0 & 9.9988\cdot 10^{-1} & 9.9785\cdot 10^{-3} & 1.7793\cdot 10^{-7}\\ 0 & 0 & 0 & -2.4758\cdot 10^{-2} & 9.9566\cdot 10^{-1} & 3.4967\cdot 10^{-5}\\ 0 & 0 & 0 & 0 & 0 & 9.0333\cdot 10^{-1} \end{array}\right], \end{array}$$

(A5) $$\begin{array}{cc} A^{5} & =\left[\begin{array}{cccccc} 1 & 9.9709\cdot 10^{-3} & 4.5486\cdot 10^{-7} & -5.1929\cdot 10^{-7} & -1.7300\cdot 10^{-9} & -1.5629\cdot 10^{-14}\\ 0 & 9.9418\cdot 10^{-1} & 8.5060\cdot 10^{-5} & -1.0375\cdot 10^{-4} & -5.1856\cdot 10^{-7} & -6.2171\cdot 10^{-12}\\ 0 & 0 & 6.6291\cdot 10^{-1} & 0 & 0 & 0\\ 0 & 0 & 0 & 9.9981\cdot 10^{-1} & 9.9783\cdot 10^{-3} & 1.7793\cdot 10^{-7}\\ 0 & 0 & 0 & -3.7387\cdot 10^{-2} & 9.9559\cdot 10^{-1} & 3.4966\cdot 10^{-5}\\ 0 & 0 & 0 & 0 & 0 & 9.0333\cdot 10^{-1} \end{array}\right], \end{array}$$

(A6) $$\begin{array}{cc} A^{6} & =\left[\begin{array}{cccccc} 1 & 9.9709\cdot 10^{-3} & 4.5486\cdot 10^{-7} & -4.6736\cdot 10^{-7} & -1.5570\cdot 10^{-9} & -1.4066\cdot 10^{-14}\\ 0 & 9.9418\cdot 10^{-1} & 8.5060\cdot 10^{-5} & -9.3378\cdot 10^{-5} & -4.6670\cdot 10^{-7} & -5.5954\cdot 10^{-12}\\ 0 & 0 & 6.6291\cdot 10^{-1} & 0 & 0 & 0\\ 0 & 0 & 0 & 9.9981\cdot 10^{-1} & 9.9783\cdot 10^{-3} & 1.7793\cdot 10^{-7}\\ 0 & 0 & 0 & -3.7387\cdot 10^{-2} & 9.9559\cdot 10^{-1} & 3.4966\cdot 10^{-5}\\ 0 & 0 & 0 & 0 & 0 & 9.0333\cdot 10^{-1} \end{array}\right], \end{array}$$

(A7) $$\begin{array}{cc} A^{7} & =\left[\begin{array}{cccccc} 1 & 9.9707\cdot 10^{-3} & 4.5690\cdot 10^{-7} & -5.2161\cdot 10^{-7} & -1.7377\cdot 10^{-9} & -1.5699\cdot 10^{-14}\\ 0 & 9.9415\cdot 10^{-1} & 8.5440\cdot 10^{-5} & -1.0422\cdot 10^{-4} & -5.2088\cdot 10^{-7} & -6.2449\cdot 10^{-12}\\ 0 & 0 & 6.6291\cdot 10^{-1} & 0 & 0 & 0\\ 0 & 0 & 0 & 9.9981\cdot 10^{-1} & 9.9783\cdot 10^{-3} & 1.7793\cdot 10^{-7}\\ 0 & 0 & 0 & -3.7387\cdot 10^{-2} & 9.9559\cdot 10^{-1} & 3.4966\cdot 10^{-5}\\ 0 & 0 & 0 & 0 & 0 & 9.0333\cdot 10^{-1} \end{array}\right], \end{array}$$

(A8) $$\begin{array}{cc} A^{8} & =\left[\begin{array}{cccccc} 1 & 9.9707\cdot 10^{-3} & 4.5690\cdot 10^{-7} & -4.6945\cdot 10^{-7} & -1.5640\cdot 10^{-9} & -1.4129\cdot 10^{-14}\\ 0 & 9.9415\cdot 10^{-1} & 8.5440\cdot 10^{-5} & -9.3796\cdot 10^{-5} & -4.6879\cdot 10^{-7} & -5.6204\cdot 10^{-12}\\ 0 & 0 & 6.6291\cdot 10^{-1} & 0 & 0 & 0\\ 0 & 0 & 0 & 9.9981\cdot 10^{-1} & 9.9783\cdot 10^{-3} & 1.7793\cdot 10^{-7}\\ 0 & 0 & 0 & -3.7387\cdot 10^{-2} & 9.9559\cdot 10^{-1} & 3.4966\cdot 10^{-5}\\ 0 & 0 & 0 & 0 & 0 & 9.0333\cdot 10^{-1} \end{array}\right], \end{array}$$

(A9) $$\begin{array}{cc} A^{9} & =\left[\begin{array}{cccccc} 1 & 9.9709\cdot 10^{-3} & 3.1840\cdot 10^{-7} & -5.1930\cdot 10^{-7} & -1.7300\cdot 10^{-9} & -1.5629\cdot 10^{-14}\\ 0 & 9.9418\cdot 10^{-1} & 5.9542\cdot 10^{-5} & -1.0376\cdot 10^{-4} & -5.1857\cdot 10^{-7} & -6.2172\cdot 10^{-12}\\ 0 & 0 & 6.6291\cdot 10^{-1} & 0 & 0 & 0\\ 0 & 0 & 0 & 9.9995\cdot 10^{-1} & 9.9787\cdot 10^{-3} & 1.7793\cdot 10^{-7}\\ 0 & 0 & 0 & -9.2220\cdot 10^{-3} & 9.9573\cdot 10^{-1} & 3.4968\cdot 10^{-5}\\ 0 & 0 & 0 & 0 & 0 & 9.0333\cdot 10^{-1} \end{array}\right], \end{array}$$

(A10) $$\begin{array}{cc} A^{10} & =\left[\begin{array}{cccccc} 1 & 9.9709\cdot 10^{-3} & 3.1840\cdot 10^{-7} & -4.6737\cdot 10^{-7} & -1.5570\cdot 10^{-9} & -1.4066\cdot 10^{-14}\\ 0 & 9.9418\cdot 10^{-1} & 5.9542\cdot 10^{-5} & -9.3383\cdot 10^{-5} & -4.6671\cdot 10^{-7} & -5.5955\cdot 10^{-12}\\ 0 & 0 & 6.6291\cdot 10^{-1} & 0 & 0 & 0\\ 0 & 0 & 0 & 9.9995\cdot 10^{-1} & 9.9787\cdot 10^{-3} & 1.7793\cdot 10^{-7}\\ 0 & 0 & 0 & -9.2220\cdot 10^{-3} & 9.9573\cdot 10^{-1} & 3.4968\cdot 10^{-5}\\ 0 & 0 & 0 & 0 & 0 & 9.0333\cdot 10^{-1} \end{array}\right], \end{array}$$

(A11) $$\begin{array}{cc} A^{11} & =\left[\begin{array}{cccccc} 1 & 9.9707\cdot 10^{-3} & 3.1983\cdot 10^{-7} & -5.2162\cdot 10^{-7} & -1.7378\cdot 10^{-9} & -1.5699\cdot 10^{-14}\\ 0 & 9.9415\cdot 10^{-1} & 5.9808\cdot 10^{-5} & -1.0422\cdot 10^{-4} & -5.2089\cdot 10^{-7} & -6.2450\cdot 10^{-12}\\ 0 & 0 & 6.6291\cdot 10^{-1} & 0 & 0 & 0\\ 0 & 0 & 0 & 9.9995\cdot 10^{-1} & 9.9787\cdot 10^{-3} & 1.7793\cdot 10^{-7}\\ 0 & 0 & 0 & -9.2220\cdot 10^{-3} & 9.9573\cdot 10^{-1} & 3.4968\cdot 10^{-5}\\ 0 & 0 & 0 & 0 & 0 & 9.0333\cdot 10^{-1} \end{array}\right], \end{array}$$

(A12) $$\begin{array}{cc} A^{12} & =\left[\begin{array}{cccccc} 1 & 9.9707\cdot 10^{-3} & 3.1983\cdot 10^{-7} & -4.6946\cdot 10^{-7} & -1.5640\cdot 10^{-9} & -1.4129\cdot 10^{-14}\\ 0 & 9.9415\cdot 10^{-1} & 5.9808\cdot 10^{-5} & -9.3800\cdot 10^{-5} & -4.6880\cdot 10^{-7} & -5.6205\cdot 10^{-12}\\ 0 & 0 & 6.6291\cdot 10^{-1} & 0 & 0 & 0\\ 0 & 0 & 0 & 9.9995\cdot 10^{-1} & 9.9787\cdot 10^{-3} & 1.7793\cdot 10^{-7}\\ 0 & 0 & 0 & -9.2220\cdot 10^{-3} & 9.9573\cdot 10^{-1} & 3.4968\cdot 10^{-5}\\ 0 & 0 & 0 & 0 & 0 & 9.0333\cdot 10^{-1} \end{array}\right], \end{array}$$

(A13) $$\begin{array}{cc} A^{13} & =\left[\begin{array}{cccccc} 1 & 9.9709\cdot 10^{-3} & 3.1840\cdot 10^{-7} & -5.1930\cdot 10^{-7} & -1.7300\cdot 10^{-9} & -1.5629\cdot 10^{-14}\\ 0 & 9.9418\cdot 10^{-1} & 5.9542\cdot 10^{-5} & -1.0376\cdot 10^{-4} & -5.1857\cdot 10^{-7} & -6.2171\cdot 10^{-12}\\ 0 & 0 & 6.6291\cdot 10^{-1} & 0 & 0 & 0\\ 0 & 0 & 0 & 9.9989\cdot 10^{-1} & 9.9785\cdot 10^{-3} & 1.7793\cdot 10^{-7}\\ 0 & 0 & 0 & -2.1851\cdot 10^{-2} & 9.9567\cdot 10^{-1} & 3.4967\cdot 10^{-5}\\ 0 & 0 & 0 & 0 & 0 & 9.0333\cdot 10^{-1} \end{array}\right], \end{array}$$

(A14) $$\begin{array}{cc} A^{14} & =\left[\begin{array}{cccccc} 1 & 9.9709\cdot 10^{-3} & 3.1840\cdot 10^{-7} & -4.6737\cdot 10^{-7} & -1.5570\cdot 10^{-9} & -1.4066\cdot 10^{-14}\\ 0 & 9.9418\cdot 10^{-1} & 5.9542\cdot 10^{-5} & -9.3381\cdot 10^{-5} & -4.6671\cdot 10^{-7} & -5.5954\cdot 10^{-12}\\ 0 & 0 & 6.6291\cdot 10^{-1} & 0 & 0 & 0\\ 0 & 0 & 0 & 9.9989\cdot 10^{-1} & 9.9785\cdot 10^{-3} & 1.7793\cdot 10^{-7}\\ 0 & 0 & 0 & -2.1851\cdot 10^{-2} & 9.9567\cdot 10^{-1} & 3.4967\cdot 10^{-5}\\ 0 & 0 & 0 & 0 & 0 & 9.0333\cdot 10^{-1} \end{array}\right], \end{array}$$

(A15) $$\begin{array}{cc} A^{15} & =\left[\begin{array}{cccccc} 1 & 9.9707\cdot 10^{-3} & 3.1983\cdot 10^{-7} & -5.2162\cdot 10^{-7} & -1.7378\cdot 10^{-9} & -1.5699\cdot 10^{-14}\\ 0 & 9.9415\cdot 10^{-1} & 5.9808\cdot 10^{-5} & -1.0422\cdot 10^{-4} & -5.2088\cdot 10^{-7} & -6.2449\cdot 10^{-12}\\ 0 & 0 & 6.6291\cdot 10^{-1} & 0 & 0 & 0\\ 0 & 0 & 0 & 9.9989\cdot 10^{-1} & 9.9785\cdot 10^{-3} & 1.7793\cdot 10^{-7}\\ 0 & 0 & 0 & -2.1851\cdot 10^{-2} & 9.9567\cdot 10^{-1} & 3.4967\cdot 10^{-5}\\ 0 & 0 & 0 & 0 & 0 & 9.0333\cdot 10^{-1} \end{array}\right], \end{array}$$

(A16) $$\begin{array}{cc} A^{16} & =\left[\begin{array}{cccccc} 1 & 9.9707\cdot 10^{-3} & 3.1983\cdot 10^{-7} & -4.6946\cdot 10^{-7} & -1.5640\cdot 10^{-9} & -1.4129\cdot 10^{-14}\\ 0 & 9.9415\cdot 10^{-1} & 5.9808\cdot 10^{-5} & -9.3798\cdot 10^{-5} & -4.6880\cdot 10^{-7} & -5.6205\cdot 10^{-12}\\ 0 & 0 & 6.6291\cdot 10^{-1} & 0 & 0 & 0\\ 0 & 0 & 0 & 9.9989\cdot 10^{-1} & 9.9785\cdot 10^{-3} & 1.7793\cdot 10^{-7}\\ 0 & 0 & 0 & -2.1851\cdot 10^{-2} & 9.9567\cdot 10^{-1} & 3.4967\cdot 10^{-5}\\ 0 & 0 & 0 & 0 & 0 & 9.0333\cdot 10^{-1} \end{array}\right], \end{array}$$

(A17) $$\begin{array}{cc} B^{1} & =\left[\begin{array}{cc} 5.7998\cdot 10^{-5} & -5.4643\cdot 10^{-5}\\ 1.6830\cdot 10^{-2} & -1.0918\cdot 10^{-2}\\ 3.0338\cdot 10^{2} & 0\\ 6.9349\cdot 10^{-6} & 3.6497\cdot 10^{-6}\\ 1.3860\cdot 10^{-3} & 1.0854\cdot 10^{-3}\\ 0 & 58.002 \end{array}\right],\;B^{2}=\left[\begin{array}{cc} 5.7998\cdot 10^{-5} & -5.4643\cdot 10^{-5}\\ 1.6830\cdot 10^{-2} & -1.0918\cdot 10^{-2}\\ 3.0338\cdot 10^{2} & 0\\ 6.9349\cdot 10^{-6} & 3.6497\cdot 10^{-6}\\ 1.3860\cdot 10^{-3} & 1.0854\cdot 10^{-3}\\ 0 & 58.002 \end{array}\right], \end{array}$$

(A18) $$\begin{array}{cc} B^{3} & =\left[\begin{array}{cc} 5.8257\cdot 10^{-5} & -5.4887\cdot 10^{-5}\\ 1.6905\cdot 10^{-2} & -1.0967\cdot 10^{-2}\\ 3.0338\cdot 10^{2} & 0\\ 6.9349\cdot 10^{-6} & 3.6497\cdot 10^{-6}\\ 1.3860\cdot 10^{-3} & 1.0854\cdot 10^{-3}\\ 0 & 58.002 \end{array}\right],\;B^{4}=\left[\begin{array}{cc} 5.8257\cdot 10^{-5} & -5.4887\cdot 10^{-5}\\ 1.6905\cdot 10^{-2} & -1.0967\cdot 10^{-2}\\ 3.0338\cdot 10^{2} & 0\\ 6.9349\cdot 10^{-6} & 3.6497\cdot 10^{-6}\\ 1.3860\cdot 10^{-3} & 1.0854\cdot 10^{-3}\\ 0 & 58.002 \end{array}\right], \end{array}$$

(A19) $$\begin{array}{cc} B^{5} & =\left[\begin{array}{cc} 5.7998\cdot 10^{-5} & -1.2162\cdot 10^{-4}\\ 1.6830\cdot 10^{-2} & -2.4301\cdot 10^{-2}\\ 3.0338\cdot 10^{2} & 0\\ 6.9348\cdot 10^{-6} & 3.6497\cdot 10^{-6}\\ 1.3859\cdot 10^{-3} & 1.0854\cdot 10^{-3}\\ 0 & 58.002 \end{array}\right],\;B^{6}=\left[\begin{array}{cc} 5.7998\cdot 10^{-5} & -1.2162\cdot 10^{-4}\\ 1.6830\cdot 10^{-2} & -2.4301\cdot 10^{-2}\\ 3.0338\cdot 10^{2} & 0\\ 6.9348\cdot 10^{-6} & 3.6497\cdot 10^{-6}\\ 1.3859\cdot 10^{-3} & 1.0854\cdot 10^{-3}\\ 0 & 58.002 \end{array}\right], \end{array}$$

(A20) $$\begin{array}{cc} B^{7} & =\left[\begin{array}{cc} 5.8257\cdot 10^{-5} & -1.2217\cdot 10^{-4}\\ 1.6905\cdot 10^{-2} & -2.4410\cdot 10^{-2}\\ 3.0338\cdot 10^{2} & 0\\ 6.9348\cdot 10^{-6} & 3.6497\cdot 10^{-6}\\ 1.3859\cdot 10^{-3} & 1.0854\cdot 10^{-3}\\ 0 & 58.002 \end{array}\right],\;B^{8}=\left[\begin{array}{cc} 5.8257\cdot 10^{-5} & -1.2217\cdot 10^{-4}\\ 1.6905\cdot 10^{-2} & -2.4410\cdot 10^{-2}\\ 3.0338\cdot 10^{2} & 0\\ 6.9348\cdot 10^{-6} & 3.6497\cdot 10^{-6}\\ 1.3859\cdot 10^{-3} & 1.0854\cdot 10^{-3}\\ 0 & 58.002 \end{array}\right], \end{array}$$

(A21) $$\begin{array}{cc} B^{9} & =\left[\begin{array}{cc} 4.0598\cdot 10^{-5} & -1.0576\cdot 10^{-5}\\ 1.1781\cdot 10^{-2} & -2.1132\cdot 10^{-3}\\ 3.0338\cdot 10^{2} & 0\\ 6.9350\cdot 10^{-6} & 3.6497\cdot 10^{-6}\\ 1.3860\cdot 10^{-3} & 1.0854\cdot 10^{-3}\\ 0 & 58.002 \end{array}\right],\;B^{10}=\left[\begin{array}{cc} 4.0598\cdot 10^{-5} & -1.0576\cdot 10^{-5}\\ 1.1781\cdot 10^{-2} & -2.1132\cdot 10^{-3}\\ 3.0338\cdot 10^{2} & 0\\ 6.9350\cdot 10^{-6} & 3.6497\cdot 10^{-6}\\ 1.3860\cdot 10^{-3} & 1.0854\cdot 10^{-3}\\ 0 & 58.002 \end{array}\right], \end{array}$$

(A22) $$\begin{array}{cc} B^{11} & =\left[\begin{array}{cc} 4.0780\cdot 10^{-5} & -1.0623\cdot 10^{-5}\\ 1.1834\cdot 10^{-2} & -2.1226\cdot 10^{-3}\\ 3.0338\cdot 10^{2} & 0\\ 6.9350\cdot 10^{-6} & 3.6497\cdot 10^{-6}\\ 1.3860\cdot 10^{-3} & 1.0854\cdot 10^{-3}\\ 0 & 58.002 \end{array}\right],\;B^{12}=\left[\begin{array}{cc} 4.0780\cdot 10^{-5} & -1.0623\cdot 10^{-5}\\ 1.1834\cdot 10^{-2} & -2.1226\cdot 10^{-3}\\ 3.0338\cdot 10^{2} & 0\\ 6.9350\cdot 10^{-6} & 3.6497\cdot 10^{-6}\\ 1.3860\cdot 10^{-3} & 1.0854\cdot 10^{-3}\\ 0 & 58.002 \end{array}\right], \end{array}$$

(A23) $$\begin{array}{cc} B^{13} & =\left[\begin{array}{cc} 4 0598\cdot 10^{-5} & -7.7558\cdot 10^{-5}\\ 1.1781\cdot 10^{-2} & -1.5496\cdot 10^{-2}\\ 3.0338\cdot 10^{2} & 0\\ 6.9349\cdot 10^{-6} & 3.6497\cdot 10^{-6}\\ 1.3860\cdot 10^{-3} & 1.0854\cdot 10^{-3}\\ 0 & 58.002 \end{array}\right],\;B^{14}=\left[\begin{array}{cc} 4.0598\cdot 10^{-5} & -7.7558\cdot 10^{-5}\\ 1.1781\cdot 10^{-2} & -1.5496\cdot 10^{-2}\\ 3.0338\cdot 10^{2} & 0\\ 6.9349\cdot 10^{-6} & 3.6497\cdot 10^{-6}\\ 1.3860\cdot 10^{-3} & 1.0854\cdot 10^{-3}\\ 0 & 58.002 \end{array}\right], \end{array}$$

(A24) $$\begin{array}{cc} B^{15} & =\left[\begin{array}{cc} 4.0780\cdot 10^{-5} & -7.7904\cdot 10^{-5}\\ 1.1834\cdot 10^{-2} & -1.5566\cdot 10^{-2}\\ 3.0338\cdot 10^{2} & 0\\ 6.9349\cdot 10^{-6} & 3.6497\cdot 10^{-6}\\ 1.3860\cdot 10^{-3} & 1.0854\cdot 10^{-3}\\ 0 & 58.002 \end{array}\right],\;B^{16}=\left[\begin{array}{cc} 4.0780\cdot 10^{-5} & -7.7904\cdot 10^{-5}\\ 1.1834\cdot 10^{-2} & -1.5566\cdot 10^{-2}\\ 3.0338\cdot 10^{2} & 0\\ 6.9349\cdot 10^{-6} & 3.6497\cdot 10^{-6}\\ 1.3860\cdot 10^{-3} & 1.0854\cdot 10^{-3}\\ 0 & 58.002 \end{array}\right], \end{array}$$

(A25) $$\begin{array}{c} W_{1}=0.01I,\;W_{2}=0.05I,\;C=\left[\begin{array}{cccccc} 1 & 0 & 0 & 0 & 0 & 0\\ 0 & 0 & 1 & 0 & 0 & 0\\ 0 & 0 & 0 & 1 & 0 & 0\\ 0 & 0 & 0 & 0 & 1 & 0\\ 0 & 0 & 0 & 0 & 0 & 1 \end{array}\right]. \end{array}$$
